# Anchor-based minimal important difference values are often sensitive to the distribution of the change score

**DOI:** 10.1007/s11136-024-03610-6

**Published:** 2024-02-06

**Authors:** Werner Vach, Franziska Saxer

**Affiliations:** 1 Department of Environmental Sciences, University of Basel, Spalenring 145, CH-4055 Basel, Switzerland; 2 Basel Academy for Quality and Research in Medicine, Basel, Switzerland; 3 Medical Faculty, University of Basel, Basel, Switzerland; 4 Novartis Institutes for Biomedical Research, Basel, Switzerland

**Keywords:** Minimal important difference, Sensitivity, Change scores, ROC curve, Predictive MID, Logistic regression

## Abstract

**Purpose:**

Anchor-based studies are today the most popular approach to determine a minimal important difference value for an outcome variable. However, a variety of construction methods for such values do exist. This constitutes a challenge to the field. In order to distinguish between more or less adequate construction methods, meaningful minimal requirements can be helpful. For example, minimal important difference values should not reflect the intervention(s) the patients are exposed to in the study used for construction, as they should later allow to compare interventions. This requires that they are not sensitive to the distribution of the change score observed. This study aims at investigating to which degree established construction methods fulfil this minimal requirement.

**Methods:**

Six constructions methods were considered, covering very popular and recently suggested methods. The sensitivity of MID values to the distribution of the change score was investigated in a simulation study for these six construction methods.

**Results:**

Five out of six construction methods turned out to yield MID values which are sensitive to the distribution of the change score to a degree that questions their usefulness. Insensitivity can be obtained by using construction methods based solely on an estimate of the conditional distribution of the anchor variable given the change score.

**Conclusion:**

In future the computation of MID values should be based on construction methods avoiding sensitivity to the distribution of the change score.

**Supplementary Information:**

The online version contains supplementary material available at 10.1007/s11136-024-03610-6.

## Plain English summary

Minimal important difference (MID) values help in interpreting changes observed in clinical trials or a care context and to assess whether a change is actually relevant. For example, if pain is scored on a rating scale from 0 (no pain) to 10 (worst imaginable pain), a reduction from 5 to 3 may be observed within a patient after some intervention, but is that relevant?

An anchor question can be used to get a better idea of this by asking the patient if they feel the same or better or worse – compared to the original state. Correlating the score with the anchor in a relevant patient sample allows determination of an MID value. There are many statistical methods for determining MID values, and there is an ongoing discussion about their adequacy.

In this paper we take a fresh look at adequacy by introducing a minimal requirement: The MID should not depend on the distribution of the score (e.g., for pain) in the patient sample used. This tries to ensure that the intervention applied in the sample cannot influence the results. This is relevant in that MID values should allow to judge any possible intervention.

We evaluate six different constructions methods for MIDs. Only one of the methods satisfies this requirement. This could be a partial explanation for the undesirable variation in MID values previously reported, as they were based on patient samples exposed to different interventions. In the future, construction methods fulfilling this minimal requirement should be preferred.

## Introduction

During the last three decades the use of PROMs (patient reported outcome measures) as patient-centric outcomes in RCTs (randomized controlled trials) or to improve the basis for communication between patients and health care providers has gained popularity [1–3]. PROMs are often implemented as summary scores, and the change from a pre-intervention assessment to a post-intervention assessment is the quantity of interest. Such changes are often hard to interpret with respect to their clinical relevance, as the scores are just expressed as an absolute number of points or standardized by statistical means, e.g., to a certain range or relative to norm data. To facilitate the interpretation of change scores, the idea of a minimal important difference (MID) has been introduced in 1989 [4]. Such a value should reflect the smallest change in the score that patients regard as important. The following years were filled by discussions about definitions, correct labelling (e.g., minimal impotent difference vs. minimal clinically important difference vs minimal important change), and construction methods [5–7]. Recently, in order to avoid the confusion about different terms, it has been suggested to focus on the more general concept of defining a “Meaningful change” [8].

Today, the most popular approach to determine MID values is based on anchor-based studies. In these studies, change scores are computed based on two consecutive measurements of the PROM of interest within a specified time interval in patients from a relevant clinical population. In addition, an anchor variable is collected close to the second measurement—or it may be constructed from the change observed in self-reported health. This variable should provide information about the magnitude of the change experienced by the patient in the construct behind the PROM of interest, e.g. pain or fatigue [7, 9]. Typically, a rating scale symmetric around "no change" is used allowing the patient to report the magnitude of an improvement or deterioration.

According to a recent systematic review [10], more than 500 studies of this type have been conducted between 1989 and 2018 suggesting more than 5000 MID values for various outcome variables. A variety of different statistical approaches have been used to determine such MID values [10, 11]. This diversity of methods to derive MID values has been characterized as a fundamental challenge to the field [7, 12–14], as different methods can yield very different values. The fundamental idea to determine the relevance of an observed change score value in a clinical or research context by comparison with an MID value is substantially flawed by this variation.

The existence of a variety of statistical approaches to define a certain quantity requires criteria to distinguish between more or less adequate approaches. This can be approached by representing the quantity of interest as a parameter in a mathematical model (or a similar framework) independent of any statistical method. Then it is possible to characterize different statistical approaches in terms of bias and precision. However, until now there is no generally accepted mathematical representation conceptualizing the term “Meaningful change”, although considering properties of the distribution of individually varying MIDs values such as the “genuine MID” [15] or measurement models [16] may represent promising approaches. The ongoing discussion about the difference between interpreting individual within-person changes and interpreting differences at the group level [7, 8, 17–19] may also indicate a need for several mathematical representations reflecting different conceptualizations of the term “Meaningful change”.

In case of lacking a consented mathematical representation of a quantity of interest, it can be useful to set up minimal requirements to statistical approaches consistent with the conceptual intentions. For example, in computing descriptive statistics measures of location can be characterized by the simple property that adding a constant *c* to all values should increase the measure also by the value *c*. This way the mean, median, and mode can be characterized as measures of location, whereas the standard deviation does not satisfy this requirement—it is a measure of variation. Such a minimal requirement does not allow to judge which measure of location is optimal. This can be only approached by setting up additional requirements.

In this paper we follow this idea and introduce a simple, minimal requirement on construction methods for MID values, which can be helpful to distinguish between more or less adequate techniques to compute MID values. Five popular techniques to construct MID values are investigated with respect to this requirement. It turns out that none of these techniques fulfil this requirement. However, a sixth approach, which has been suggested implicitly in previous research, fulfils this requirement.

## MID values should not be sensitive to the distribution of the change score in an anchor-based MID study

MID values should reflect the magnitude of a change score at which patients tend to experience such a change as important. This value may depend on patient characteristics such as disease type, disease severity, disease duration, age, gender or education [20–22]. The choice of a relevant and well-described patient population in an anchor-based MID study is hence essential to allow the selection of adequate MID values to assist in the interpretation of change scores.

Anchor-based MID studies may not only differ in the patient population used, but also with respect to the intervention(s) the patients are exposed to. There may be no active intervention (“usual care”), some standard intervention, an experimental intervention, or a mixture of different interventions. Whereas it is natural that the MID value constructed depends on characteristics of the patients included, the value should not depend on the intervention(s). This is at least necessary if MID values are later used to compare interventions, e.g., by using MID values as cut points in a responder analysis or by comparing them with observed average intervention effects. However, insensitivity to the intervention(s) is already a consequence of the conceptual definition of MID values, relating them to the experience of patients independent of any specific intervention.

If MID values should not depend on the intervention(s) the patients are exposed to, they have to be insensitive to the distribution of the change score observed in the MID study, as different interventions will typically imply different distributions of the change scores depending on the effectiveness of the intervention. Hence insensitivity to the distribution of the change score is a meaningful requirement on construction methods for MID values based on anchor-based MID studies.

The requirement of insensitivity to the distribution of the change score can be also motivated by the logic of anchor-based MID studies. Such studies try to explore how patients tend to translate an empirically observed change score into an experienced change. These individual translation processes are logically in no way related to the population distribution of the change scores in the specific study. Using two patient populations performing the same translation processes but differing in the distribution of the change score should results in very similar MID values.

A more formal definition of the insensitivity to the distribution of the change score is given at the end of the next section.

## Two perspectives on the data generated by an anchor-based MID study

**Fig. 1:**
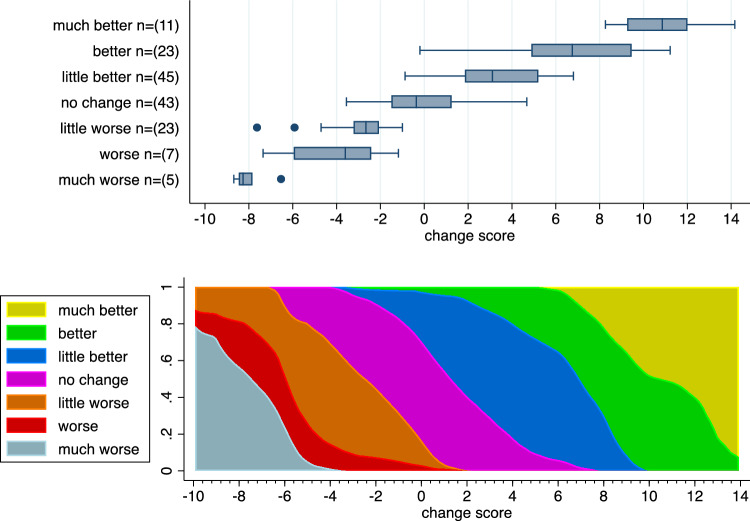
Visualization of the data from a hypothetical anchor-based MID study. Upper part: Boxplots of the change score stratified by the anchor variable. Lower part: Stacked area plot for the conditional distribution of the anchor variable given the change score. Details on the computation of this plot are given in Supplementary Appendix 1

Traditionally, the analysis of anchor-based MID studies is based on considering the distribution of the change score in subgroups of patients defined by specific anchor values, i.e., the conditional distribution of the change score given the anchor values. This perspective is reflected in visualizations of the type shown in the upper part of Fig. 1, and such visualizations have been recommended to present the data of MID studies [7]. An alternative perspective is to consider the conditional distribution of the anchor variable given the change score values. This perspective is reflected in the lower part of Fig. 1, allowing to read off for each potential value $c$ of the change score an estimate of the distribution of the anchor variable (in terms of probability of each anchor response).

**Fig. 2:**
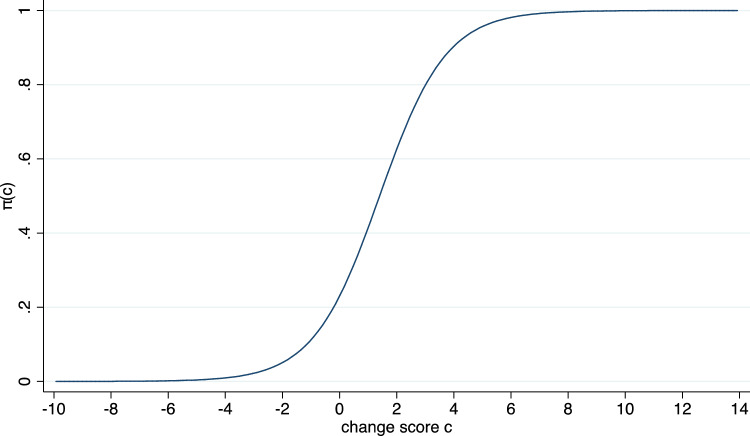
An estimate of the function $$\pi (c)$$ based on fitting a logistic regression model to the data set considered in Fig. 1. $$\pi (c)$$ reflects the probability that a patient with a change score value of $$ c$$ reported an improvement

Of particular interest are the values of the anchor variable indicating an improvement, i.e., in the example of Fig. 1 “little better”, “better”, or “much better”. In addition, the function

$$\pi (c)= P(Patient\,reported\,an\,improvement | Patient\,experienced\,a\,change\,score\,c)$$

is of interest and plays a central role in recently suggested methods to compute MID values [15, 23, 24]. An estimate of $\pi (c)$ can be obtained by fitting a logistic regression model to the binary indicator variable “Patient reported an improvement” with the change score as single covariate. The corresponding estimate of $\pi (c)$ for the data set considered in Fig. 1 is shown in Fig. 2.

The second perspective also allows us to give a more formal definition of the insensitivity to the distribution of the change score of a construction method: If two anchor-based studies share the relationship between the anchor variable and the change score as depicted by the conditional distribution of the anchor variable given the change score, the application of the construction method should result in similar values and on average the expected value of the difference should be 0—also if the distribution of the change score differs between the studies*.*

As mentioned in the Introduction, insensitivity to the distribution of the change score is a minimal requirement. This means that we judge adequacy of construction methods solely in terms of this insensitivity. We do not consider bias or precision with respect to a `true’ MID value, i.e., we do not contribute to the question whether construction methods result in reasonable estimates of a meaningful change.

## Some construction methods for MID values

In this paper, we consider six different methods to construct MID values. The first three methods have a long tradition in the field of anchor-based MID studies, and according to [10] they cover 89% of all computations of MID values based on change scores. These three methods take the more traditional view on the data of an anchor-based MID study shown in the upper part of Fig. 1. The next two methods are recent developments reflecting a methodological shift towards the perspective represented in the lower part of Fig. 1. They use an estimate of $\pi (c)$ to derive at MID values. The final method considered simply reads off the MID value from an estimate of $\pi (c).$ In the sequel we describe the techniques used in the construction methods, but we do not discuss in depth the question of differences in the interpretation of the values obtained.

### Mean change

MID_mean_ is defined as the mean change score observed in the subgroup of patients with a minimal improvement. This is the most frequently used method in anchor-based MID studies [10]. In the example of Fig. 1 this is the mean change score in those patients reporting «little better».

### ROC-curve based MID values

The problem to determine an MID value can be seen as the task to optimally distinguish between the two states “improved” or “not improved”, i.e., as the problem to “diagnose” this status of a subject based on the observed change score. In such a diagnostic setting, it is common to visualize the relationship between the continuous change score and the two states by an ROC curve. Deriving MID values from the ROC curve is the second-most frequently used approach [10]. However, different rules to arrive at a classification rule based on the ROC curve have been used. In this paper, we will consider the rule to determine a cut point for the change score by maximizing the Youden index [25], which is equivalent to maximizing the average of sensitivity and specificity. This optimal cut point defines MID_Youden_. For a specific cut point, sensitivity and specificity depict an aspect of the distribution of the change score in the groups of “improved” and “not improved” patients, respectively. Hence this approach is related to the traditional perspective.

### Mean difference

MID_diff_ is defined as the difference in mean change score between the patients reporting a minimal improvement and those reporting no change. This is the third-most frequently used method in MID studies [10].

### The predictive MID

Terluin et al. [23] suggested to apply a logistic regression model to the binary improvement indicator variable (improved vs. non-improved) with the change scores as covariate. This results in an estimate of the function $\pi \left(c\right)$. MID_predictive_ is then defined as the change score value $c,$ for which the post odds according to the model are equal to the pre odds neglecting any information on the change scores. This means that $\pi ($ MID_predictive_) is equal to the relative frequency of patients reporting an improvement.

### The adjusted MID

Terluin et al. [15] suggested to use MID_predictive_ as a starting point to approximate the genuine MID. The genuine MID assumes that each patient has its own individual MID value, and that a patient reports an improvement if the experienced change score is above her or his individual MID value. The genuine MID is then defined as the population mean of the individual MID values. Terluin et al. [15] conducted a large-scale simulation study to investigate the relationship between MID_predictive_ and the genuine MID and derived a correction formula defining MID_adjust_ (Supplementary Appendix 2).

### MID_50_

Once an estimate of the function $\pi \left(c\right)$ has been derived by fitting a logistic regression model, we can determine for each potential value $c$ of the change score the probability that a patient experiencing this change score reported an improvement. Especially we can determine the change score at which half of the patients reported an improvement. The simple interpretation of this change score value qualifies it to be considered as an MID value. Consequently, we define MID_50_ = ${\pi }^{-1}(0.5)$ with ${\pi }^{-1}$ denoting the inverse of the function $\pi \left(.\right)$.

MID_50_ is closely related to the genuine MID. Assuming independence between observed change scores and the individual MID values, MID_50_ is an estimate of the median of the distribution of the individual MID values (Supplementary Appendix 3). If this distribution is symmetric, MID_50_ aims at estimating the genuine MID and is hence close in spirit to MID_adjust_. To the best of our knowledge, MID_50_ has not been suggested explicitly as an MID value in the literature but has been discussed implicitly (Supplementary Appendix 4).

### A note on within-person changes and between-group difference

As mentioned in the introduction, in interpreting MID values a distinction between within-person changes and between-group differences may matter. However, this distinction is not relevant for this study. We simply aim at determining whether construction methods are sensitivity to the distribution of the change score or not—regardless of any intended interpretation of MID values.

## Quantifying the sensitivity to the distribution of the change score

Insensitivity to the distribution of the change score as introduced in Sects. "MID values should not be sensitive to the distribution of the change score in an anchor-based MID study" and "Two perspectives on the data generated by an anchor-based MID study" is a qualitative property of a construction method: either a method has this property, or it does not have this property. However, the lack of insensitivity, i.e. sensitivity, has also a quantitative component: the degree of sensitivity may vary from construction method to construction method. This quantitative aspect was investigated in a simulation study for the six construction methods for MID values introduced in the previous section.

**Fig. 3:**
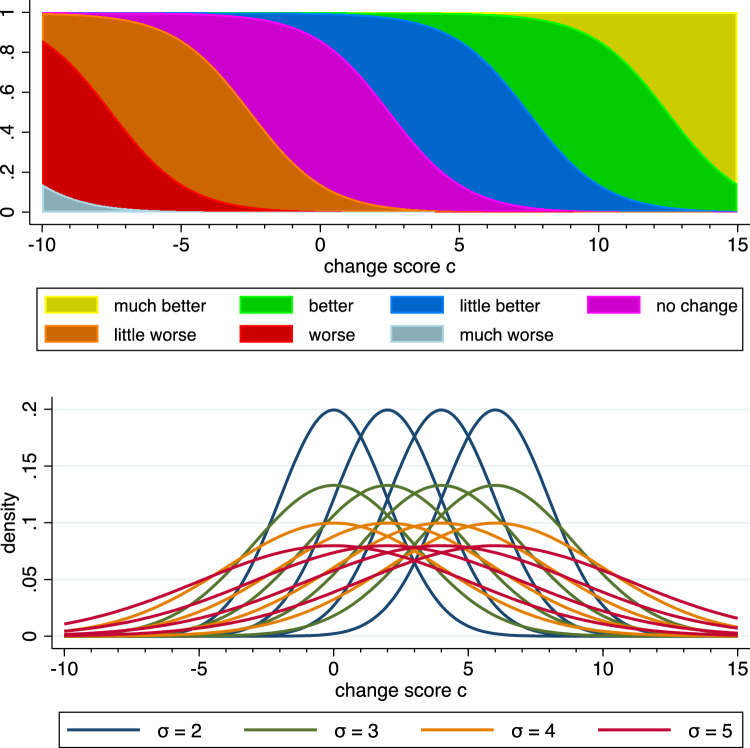
Set-up of the simulation study. The upper part shows the conditional distribution of the anchor variable given the change score value. The lower part shows the sixteen distributions of the change score considered. The mean values $\upmu$ are 0, 2, 4, or 6, respectively. A formal description of the set-up is provided in Supplementary Appendix 5

The setting of the simulation study considers one patient population, which is characterized by a specific relationship between the change score experienced by a patient and the patient’s response defining the anchor variable. The relationship assumed is depicted in the upper part of Fig. 3, i.e., a specific choice for the conditional distribution of the anchor variable given the change score values. A single anchor-based MID study was generated by drawing change scores from a normal distribution and subsequently drawing the anchor variable from the specified conditional distribution. For the normal distribution different choices of the mean $\mu$ and the standard deviation $\sigma$ were considered as indicated in the lower part of Fig. 3, and a sample size of 123 was assumed. In each simulated study the six MID values outlined above can be computed. Averaging these values over 1000 simulated studies inform us about the magnitude of the MID values to be expected.

**Fig. 4:**
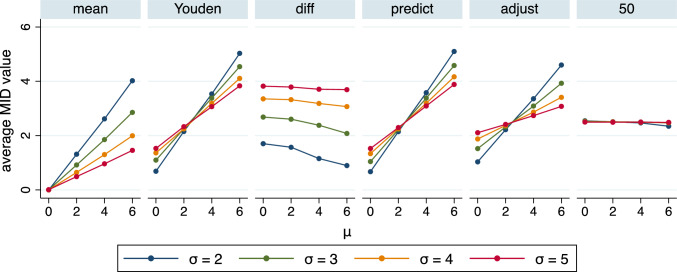
Results of the simulation study. The average MID value is shown for each of the sixteen choices of the change score distribution and for each of the six construction methods. The exact numerical values can be found in Supplementary Appendix 7

Figure 4 shows the results of this simulation study. MID_50_ is the only construction method which is insensitive to the choice of the distribution: on average always the same MID value of about 2.5 is obtained. This value coincides with the upper part of Fig. 3, indicating that at a change score of 2.5 half of the patients tend to report an improvement. The average values of MID_mean_, MID_Youden_, MID_predict_, and MID_adjust_ all depend on the mean value of the distribution of the change score, and MID_diff_ depends on the standard deviation. The dependence is most pronounced for MID_Youden_ and MID_predict_. If the change score has a moderate variation corresponding to a standard deviation of 2, moving the mean change score from 0 to 6 implies average MID values moving from less than 1 to more than 5. If the change score has a higher variation the range reduces but for a standard deviation of 5 the range still extends from less than 2 up to 4. The sensitivity to the distribution of the change score is slightly less pronounced for MID_mean_, with ranges from 0 to 4 or from 0 to about 1.5, respectively. MID_adjust_ is even less sensitive with ranges from about 1 to about 4.5 or from about 2 to about 3. The average values of MID_diff_ are more sensitive to the variation of the change scores than to the mean value of the distribution of the change score and range from 1 to 4. Further aspects of the results of this simulation study are discussed in Supplementary Appendix 6.

## Establishing insensitivity

Although the simulation study could only consider one specific choice of the conditional distribution, this is sufficient to conclude that five of the six construction methods do not fulfil the requirement of insensitivity to the distribution of the change score, as this requirement should hold for all choices. Moreover, the degree of sensitivity observed cannot be regarded as negligible—a result which could be confirmed in two further simulation studies reported in Supplementary Appendices 8 and 9.

The simulation study suggests that MID_50_ is a construction method satisfying the requirement of insensitivity. However, this is not a proof, as only one constellation was considered. There is a need for a formal, general argument: the insensitivity of MID_50_ to the distribution of the change score is a simple consequence of using the function $\pi (c)$ to define the MID value. $\pi (c)$ describes the conditional distribution of the improvement indicator given the change score, and by definition a conditional distribution given the change score does not depend on the distribution of the change score. Hence the insensitivity to the distribution of the change score holds for any MID construction method based solely on an unbiased estimate of $\pi (c)$, and this holds for any distribution of the change score and any conditional distribution—not only for those depicted in Fig. 3.

## Discussion

Insensitivity to the distribution of the change score is a meaningful minimal requirement on construction methods for MID values derived from an anchor-based MID study. Our investigation shows that many construction methods for MID values do not fulfil this requirement. This questions their usefulness in current practice. It may also partially explain why systematic reviews often find a wide range of MID values for the same instrument [7, 26–30]. Insensitivity can be obtained by basing the computation of MID values solely on an estimate of the conditional distribution of the anchor variable given the change score, and in particular on an estimate of the function $\pi (c)$. It is not sufficient that construction methods just make use of such an estimate, as illustrated by MID_predict_.This principle is based on relating an estimate of $\pi (c)$ to the overall prevalence of reporting an improvement, and hence it is not solely based on this estimate.

The sensitivity of MID_predict_ to the prevalence of improvement has been the motivation for the development of MID_adjust_ [15], and it has been reported also for other construction methods [31, 32]. The sensitivity to the prevalence is closely related to the sensitivity to the distribution of the change score: Keeping the conditional distribution of the anchor variable given the change score fixed, an increase in the mean value of the distribution of the change score implies an increase in the prevalence of reporting an improvement. However, insensitivity to the distribution of the change score is a broader requirement. This is illustrated by MID_diff_, which is rather insensitive to changes in the mean value, but sensitive to changes in the variation. Recently, the sensitivity to the prevalence has been reported as a general challenge to anchor-based approaches [33]. However, construction methods such as MID_50_ can solve this issue.

The insensitivity to the distribution of the change scores defines a minimal requirement reflecting a necessary condition for the usefulness of a construction method for MID values. It does not define a sufficient condition. For example, fixing the MID to the value 10 is a rather meaningless construction method, but one that is insensitive to the distribution of the change score by definition. The class of construction methods satisfying the necessary condition may be rather large. There are many different statistical methods to derive at an estimate of $\pi (c)$, the value 50% can be exchanged by other values, and there may be construction methods depending on the conditional distribution of the anchor variable in a very complex manner. Hence additional requirements are necessary to select from all these methods those allowing to determine a meaningful change from a conceptual point of view. Developing frameworks that allow the definition of a `true’ MID value can be a basic step forward allowing the popular distinction between estimands and estimates [34]. The assumption of individual MID values and the definition of the genuine MID provides an example of such a framework. More complicated frameworks can be defined by additionally taking measurement error in the change score and the anchor into account [11, 35]. The usefulness of MID_50_ within such frameworks has still to be examined by dedicated explorations of bias and precision in recovering a `true’ MID value before any recommendation can be made. In the context of this paper MID_50_ simply demonstrates the existence of construction methods satisfying the insensitivity requirement.

We mentioned in Sect. "Some construction methods for MID values", that the insensitivity requirement can be applied regardless of the intended interpretation of the change score. With respect to the six construction methods considered, especially the mean difference method has been regarded suitable to estimate between-group differences [17, 19]. Indeed, this method showed a different type of insensitivity than the other methods: Only the variance of distribution of the change score does matter but not the mean. This may support the suitability for between-group differences.

It should be noted that the function $\pi (c)$ cannot only be used to define MID values such as MID_50_. It can also be used to improve the interpretation of single change score values $c$ by computing $\pi (c$). This is the probability that a randomly chosen patient will report an improvement if the patient experiences this change score. This can help to interpret single individually observed change scores. In addition, this transformation can also be applied to an observed treatment difference $\Delta$ at the group level. Then $\pi (\Delta$) is the probability that a randomly chosen patient will report an improvement if the patient experiences a change score equal to $\Delta$. As pointed out in the introduction, it is debatable whether treatment differences at the group level should be handled in the same way as individual change score values. However, it can still be argued that the transformation provides more information than the simple comparison of $\Delta$ with MID values frequently found in the literature. For example, if $\Delta$ is twice the MID_50_, $\pi (\Delta$) can be still 70% or 90%, depending on how steep the function $\pi (c)$ is.

Recent developments to construct MID values are based on item response theory (IRT). They use as input not the change score values, but observations of the single items at both time points [31, 32]. For these construction methods insensitivity to the joint distribution of these items should be required. If the MID value is derived solely from the fitted IRT model, this probably implies insensitivity, as an IRT model describes the conditional distribution of the anchor variable given the items.

## Conclusion

MID values should not be sensitive to the distribution of the change score in an anchor-based MID study. Many established construction methods for MID values do not fulfil this requirement. Focusing on construction methods based solely on the conditional distribution of the anchor variable given the change score can ensure this requirement and may hence be preferred in future.

### Supplementary Information

Below is the link to the electronic supplementary material.

Supplementary file1 (PDF 573 KB)

Supplementary file1 (DO 9 KB)

## Data Availability

Not applicable. The code of the simulations performed is available as a supplement.
