# Supplementary material for: Anchor-based minimal important difference values are often sensitive to the distribution of the change score
Source: Qual Life Res. 2024 Feb 6;33(5):1223–32. doi: 10.1007/s11136-024-03610-6 (PMC11045581; doi:10.1007/s11136-024-03610-6)
Supplement: Supplementary file 1 — Supplementary file1 (PDF 573 KB) [file 11136_2024_3610_MOESM1_ESM.pdf]

Supplementary Material referring to the following article to be published in Quality of Life Research:

Anchor-based minimal important difference values are often sensitive to the distribution of the change score

Werner Vach[a,b], Franziska Saxer[c,d]

[a]Department of Environmental Sciences, University of Basel, Basel, Switzerland, email: werner.vach@unibas.ch

[b]Basel Academy for Quality and Research in Medicine, Basel, Switzerland

[c]Medical Faculty, University of Basel, Basel, Switzerland, email: franziska.saxer@unibas.ch

[d]Novartis Institutes for Biomedical Research, Basel, Switzerland

Corresponding author: Werner Vach, Department of Environmental Sciences, University of Basel, Spalenring 145, CH-4055 Basel, Switzerland, email: werner.vach@unibas.ch, phone: +41 61 285 1034

ORCID:
Werner Vach: 0000-0003-1865-8399
Franziska Saxer: 0000-0003-4153-6795

Appendix 1: Computations behind the stacked area plot

Constructing an empirical, model-free estimate of the distribution of the anchor variable A given the value c of the change score C is challenging, as the change score is a continuous variable. Consequently, the application of smoothing procedures is necessary.

According to Bayes theorem, the conditional distribution of A given $C = c$ can be expressed as

$$P(A = a|C = c) = \frac{f(c|A = a)\,P(A = a)}{\sum_{a'} f(c|A = a')\,P(A = a')}$$

with the summation ranging over all possible values of the anchor variable. Empirical estimates of $f(c|A = a)$ and of $P(A = a)$ can be inserted into this formula.

In our example, estimates of $f(c|A = a)$ were obtained by using a kernel density estimate in the subgroup of patients with $A = a$. For $P(A = a)$ the empirical relative frequencies were used as estimates. The resulting values of $P(A = a|C = c)$ were visualized in a stacked area plot.

Appendix 2: The formula for $\mathrm{MID}_{\mathrm{adjust}}$

Assuming a normal distribution for the individual MID values and independence between the change score values and the individual MID values, Terluin et al. [15] conducted a large-scale simulation study to investigate the relationship between $\mathrm{MID}_{\mathrm{predictive}}$ and the genuine MID. From this simulation study they derived the formula

$$\mathrm{MID}_{\mathrm{adjust}} = \mathrm{MID}_{\mathrm{predictive}} - S \times log\ odds\ (p)$$

with p denoting the prevalence of reporting an improvement and

$$S = 0.09 \times SD_{change} + 0.103 \times SD_{change} \times r$$

with SD_{change} denoting the standard deviation of the change scores and r denoting the Pearson correlation between the change score and the binary improvement indicator variable.

Appendix 3: The relationship between $\pi(c)$ and the distribution of individual MID values

With C denoting the change score and MID_{ind} the individual MID value, $\pi(c)$ can be expressed as

$$\pi(c) = P(C > MID_{ind} \mid C = c) = P(MID_{ind} < c \mid C = c).$$

Assuming that C is independent of MID_{ind}, we have

$$P(MID_{ind} < c \,|C = c) \;=\; P(\,MID_{ind} < c\,) \;=\; F^{MID_{ind}}(c)$$

i.e., $\pi(c)$ is identical to the distribution function of MID_{ind}.

Consequently, under this assumption MID_{50} is equal to $F^{MID_{ind}}(0.5)$, i.e., the median of the distribution of MID_{ind}.

However, it is not unlikely that there is a positive association between the individual MID values MID_{ind} and the change score C, as both may be correlated to the patient's enthusiasm to react to changes in the true health status. Those patients, who translate already small changes in the true health status to large change scores may be also those who require a higher level to regard a change as relevant, as they know (implicitly) their response behaviour.

It is important to note that the interpretation of MID_{50} as the change score value at which half of the patients report an improvement is valid independent of any assumptions between the observed change scores and the individual MID values.

Appendix 4: The relationship between MID_{50} and existing methods

Beside MID_{adjust} several methods have been suggested to approximate the genuine MID, which hence can be seen as attempts to approximate MID_{50} [24, 31, 32]. In particular, Bjorner et al. [31] mention explicitly that the genuine MID can be characterized by the level of health improvement at which the probability of a meaningful improvement equals the probability of no meaningful improvement, i.e. 0.5. The method developed in [31] aims at estimating this level. However, it is based on a longitudinal item response model using the single items constituting the score as input instead of using the change score itself directly.

Appendix 5: Formal description of the simulation set up

The simulation study is based on generating data for a single anchor-based MID study, i.e., values of the change score C and values of the anchor variable A. The values of C are drawn from a normal distribution with mean μ and standard deviation σ. Given the values of the change score C , the anchor variable A is draw from an ordinal logit model, which can be described by

$$P(A = a|C = c) = \Lambda(\kappa_a - 0.4 \times c) - \Lambda(\kappa_{a-1} - 0.4 \times c)$$

with a ranging from 1 to 7 and $\kappa_0 = -\infty, \kappa_1 = -5, \kappa_2 = -3, \kappa_3 = -1,, \kappa_4 = 1, \kappa_5 = 3, \kappa_6 = 5$, and $\kappa_7 = \infty$. The values 1 to 7 represent the levels much worse, worse, little worse, no change, little better, better, and much better and $\Lambda(x) = 1/\left(1 + exp\left(-\sqrt{\pi^2/3}\,x\right)\right)$ is the distribution function of a logistic distribution with mean 0 and variance 1.

The sample size for a single MID study is chosen as 123. For one choice of μ and σ, 1000 studies are generated. For each of the 6 methods the MID value is computed, and the

average is taken over the 1000 studies. To judge the accuracy of this mean in approximating the expected value, the standard error of the mean is computed, too.

Sixteen combinations of values for μ and σ are considered. The values of μ are 0, 2, 4, and 6, and the values of σ are 2, 3, 4, and 5. The expected Spearman correlation between A and C ranges from 0.56 for σ=2 to 0. 85 for σ=5, and the prevalence values of reporting an improvement range from 0.21 to 0.87.

The simulation study was performed with Stata 15.1. The computer code is available in an additional Supplementary File.

Appendix 6: Further aspects of the results of the simulation study

MID_{Youden} and $MID_{predict}$ give very similar results in this simulation study. This coincides with the derivation of $MID_{predict}$ by Terluin et al. [23], introducing $MID_{predict}$ as a conceptually similar, but more precise substitute for MID_{Youden}.

MID_{adjust} is clearly less sensitive than $MID_{predict}$. This corresponds to the considerations to regard MID_{adjust} as an approach to approximate MID_{50}. This approximation seems to work rather well if the variation of the change scores is high.

The insensitivity of MID_{diff} to the mean value of the distribution of the change score can be explained by the construction method aiming at the difference in mean change score between two adjacent subgroups of patients defined by the anchor variable. A change in the mean of the change scores affects both groups in a similar manner.

Appendix 7: Numerical results of the simulation study

The following table shows the mean of the MID values (upper number) and the corresponding standard error of the mean (lower number) based on the 1000 simulated studies for each method and the 16 combinations of μ and σ.

.

	sigma and mu							
	2				3			
method	0	2	4	6	0	2	4	6
50	2.544	2.510	2.467	2.345	2.532	2.510	2.500	2.465
	0.017	0.010	0.012	0.027	0.013	0.011	0.012	0.018
Youden	0.686	2.151	3.535	5.030	1.097	2.254	3.381	4.540
	0.018	0.016	0.017	0.021	0.021	0.018	0.019	0.022
adjust	1.032	2.218	3.357	4.601	1.520	2.317	3.089	3.928
	0.007	0.005	0.006	0.009	0.009	0.007	0.008	0.011
diff	1.702	1.569	1.153	0.895	2.683	2.603	2.379	2.078
	0.012	0.020	0.042	0.085	0.015	0.021	0.037	0.067
mean	-0.003	1.313	2.617	4.022	0.004	0.918	1.852	2.854
	0.006	0.007	0.009	0.015	0.008	0.009	0.011	0.016
predict	0.670	2.144	3.580	5.099	1.043	2.220	3.381	4.582
	0.006	0.005	0.005	0.008	0.008	0.007	0.008	0.010

method	sigma and mu 4: 0	2	4	6	5: 0	2	4	6
50	2.504	2.501	2.488	2.478	2.498	2.492	2.496	2.487
	0.013	0.011	0.012	0.015	0.014	0.013	0.013	0.015
Youden	1.363	2.250	3.187	4.107	1.529	2.330	3.065	3.834
	0.023	0.021	0.021	0.024	0.025	0.023	0.024	0.026
adjust	1.876	2.373	2.863	3.410	2.107	2.413	2.737	3.077
	0.011	0.009	0.010	0.012	0.012	0.011	0.012	0.013
diff	3.353	3.324	3.185	3.069	3.821	3.789	3.708	3.691
	0.017	0.021	0.029	0.047	0.019	0.022	0.028	0.039
mean	-0.001	0.643	1.302	1.992	0.007	0.488	0.961	1.456
	0.010	0.010	0.013	0.016	0.012	0.012	0.014	0.016
predict	1.335	2.260	3.189	4.167	1.524	2.296	3.092	3.885
	0.010	0.008	0.009	0.011	0.011	0.010	0.011	0.012

Appendix 8: Set-up and results of a second simulation study

In the second simulation study, the anchor variable is drawn from an ordinal probit model instead of an ordinal logit model, i.e.

$$P(A = a|C = c) = \Phi(\kappa_a - 0.4 \times c) - \Phi(\kappa_{a-1} - 0.4 \times c)$$

with $\Phi(x)$ denoting the distribution function of a standard normal distribution. The thresholds values are now chosen as $\kappa_0 = -\infty, \kappa_1 = -5, \kappa_2 = -3.5, \kappa_3 = -1,, \kappa_4 = 1, \kappa_5 = 2, \kappa_6 = 5$, and $\kappa_7 = \infty$. This means that they are no longer equidistant as in the original simulation.

In addition, the change scores are now drawn from a weighted mixture of two normal distributions with weights 0.75 and 0.25 and mean values μ-0.5 × σ and μ + 1.5 × σ and equal variance. The variance is chosen such that the overall standard deviation is equal to σ. The set-up of the second simulation study is depicted in Figure A1.

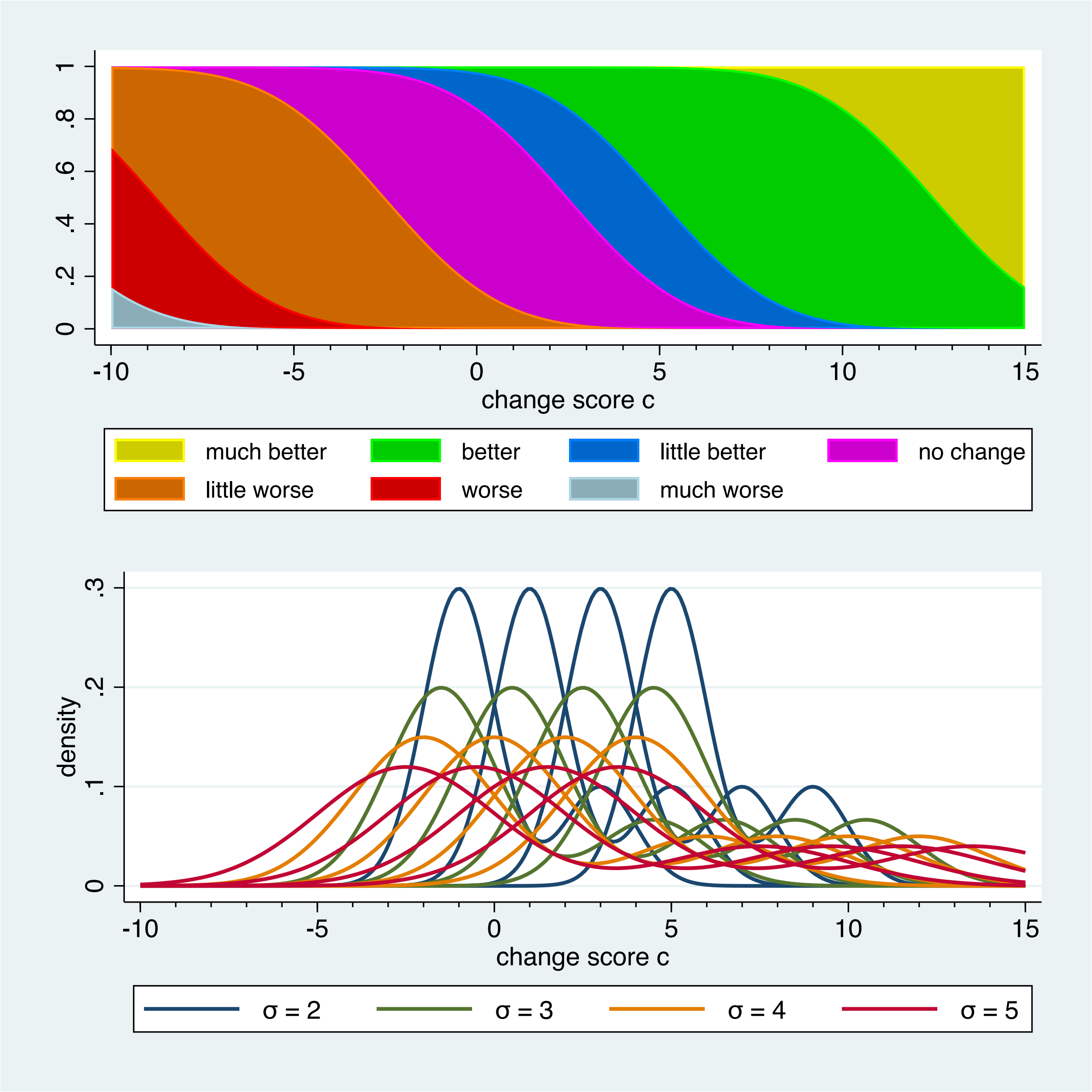

Figure A1: Set-up of the second simulation study. The upper part shows the conditional distribution of the anchor variable given the change score value. The lower part shows the sixteen distributions of the change score considered. The mean values μ are 0, 2, 4, or 6, respectively.

The results of the second simulation study are shown in Figure A2. Similar to Figure 4, a substantial variation of the average MID values in dependence on μ and σ can be observed. There is one basic difference for those construction methods showing a relationship of the average values to μ and σ: The relationship is now always non-linear.

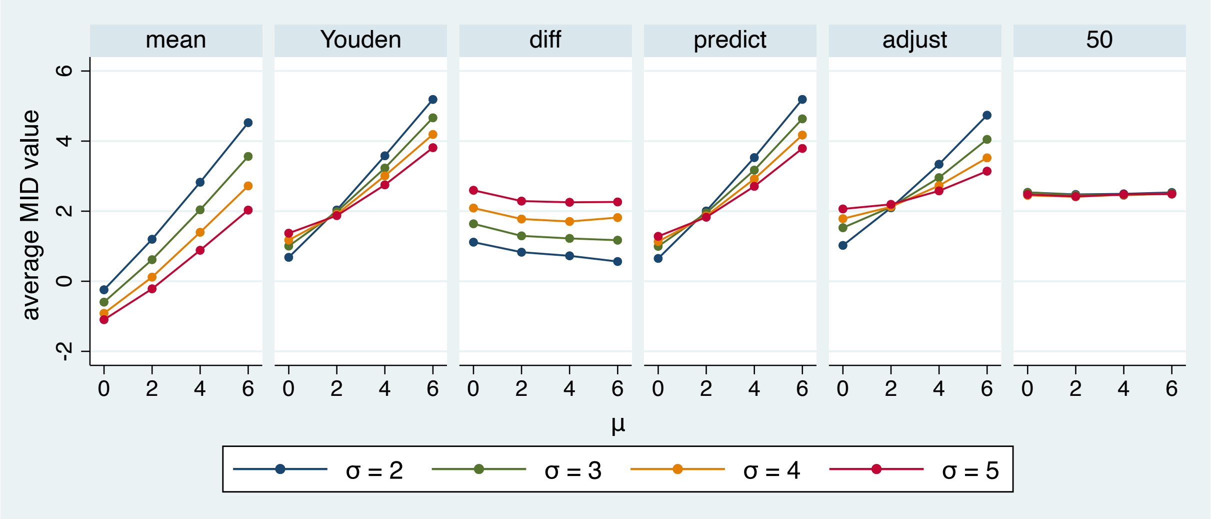

Figure A2: Results of the second simulation study. The average MID value is shown for each of the sixteen choices of the change score distribution and for each of the six construction methods.

This second simulation study indicates that a slight misspecification of the model (probit model for data generation and logistic model for model fitting) and an asymmetric distribution of the change score have a limited effect on the simulation results. With respect to the choice of the thresholds, an impact could be only expected for MID_{mean} and MID_{diff}, as all other construction methods only depend on the binary indicator variable "Patient reported an improvement", and not on the full distribution of the anchor variable.

Appendix 9: Set-up and results of a third simulation study

The third simulation study differs from the original one with respect to the regression coefficient in the ordinal logit model, which has now the values 0.2 instead of 0.4. In addition, the thresholds are chosen as $\kappa_0 = -\infty, \kappa_1 = -2.5, \kappa_2 = -1.5, \kappa_3 = -0.5,, \kappa_4 = 0.5, \kappa_5 = 1.5, \kappa_6 = 2.5$, and $\kappa_7 = \infty$. The set-up of the second simulation study is depicted in Figure A3. The expected Spearman correlation between A and C ranges from 0.36 for σ=2 to 0. 68 for σ=5, and the prevalence values of reporting an improvement range from 0.31 to 0.70.

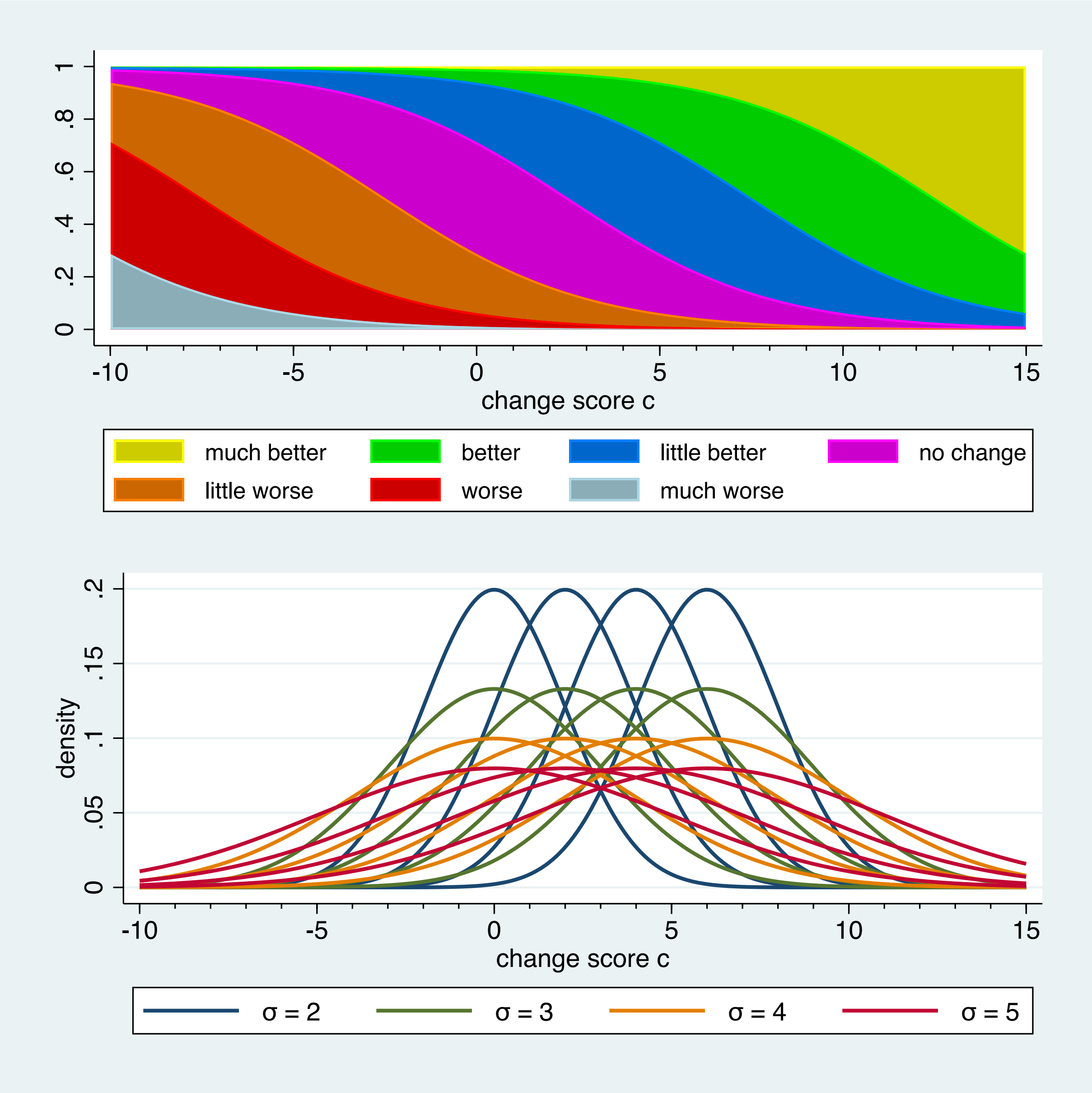

Figure A3: Set-up of the third simulation study. The upper part shows the conditional distribution of the anchor variable given the change score value. The lower part shows the sixteen distributions of the change score considered. The mean values μ are 0, 2, 4, or 6, respectively.

The results of the third simulation study are shown in Figure A4. They are very similar to those presented in Figure 4. Note that in Figure A4 the median and not the average value of the MID values is reported. This takes into consideration, that within the simulation some datasets show a correlation close to 0 which may result in rather implausible estimates of the MID value. However, this happens not very frequently, such that the median is not affected.

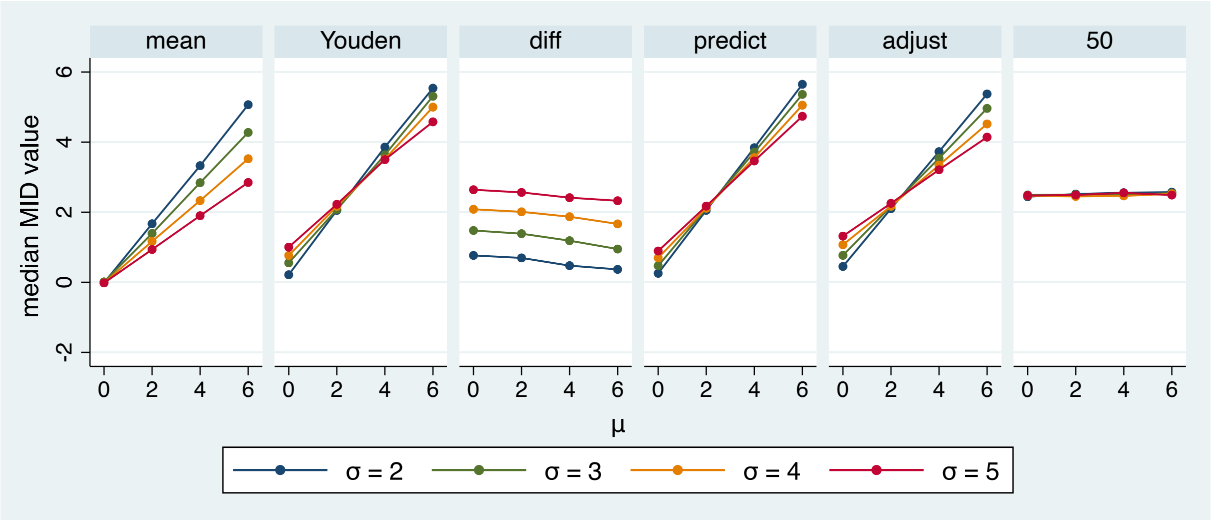

Figure A4: Results of the third simulation study. The average MID value is shown for each of the sixteen choices of the change score distribution and for each of the six construction methods.

The third simulation study indicates that the degree of insensitivity of the five construction methods does not decrease with decreasing degree of association between the change score and the anchor variable. In addition, the insensitivity of MID_{50} remains.
